# A single-centre prospective evaluation of left bundle branch area pacemaker implantation characteristics

**DOI:** 10.1007/s12471-022-01679-7

**Published:** 2022-04-05

**Authors:** L. I. B. Heckman, J. G. L. M. Luermans, M. Jastrzębski, B. Weijs, A. M. W. Van Stipdonk, S. Westra, D. den Uijl, D. Linz, M. Mafi-Rad, F. W. Prinzen, K. Vernooy

**Affiliations:** 1 Department of Physiology, Cardiovascular Research Institute Maastricht, Maastricht University, Maastricht, The Netherlands; 2 Department of Cardiology, Cardiovascular Research Institute Maastricht, Maastricht University Medical Centre+, Maastricht, The Netherlands; 3 Department of Cardiology, Radboud University Medical Centre, Nijmegen, The Netherlands; 4 First Department of Cardiology, Interventional Electrocardiology and Hypertension, Jagiellonian University Medical College, Kraków, Poland; 5 Department of Cardiology, Marienhospital Aachen, Aachen, Germany

**Keywords:** Left bundle branch pacing, Bradycardia pacing, Cardiac resynchronisation therapy

## Abstract

**Background:**

Left bundle branch area pacing (LBBAP) has recently been introduced as a physiological pacing technique with synchronous left ventricular activation. It was our aim to evaluate the feasibility and learning curve of the technique, as well as the electrical characteristics of LBBAP.

**Methods and results:**

LBBAP was attempted in 80 consecutive patients and electrocardiographic characteristics were evaluated during intrinsic rhythm, right ventricular septum pacing (RVSP) and LBBAP. Permanent lead implantation was successful in 77 of 80 patients (96%). LBBAP lead implantation time and fluoroscopy time shortened significantly from 33 ± 16 and 21 ± 13 min to 17 ± 5 and 12 ± 7 min, respectively, from the first 20 to the last 20 patients. Left bundle branch (LBB) capture was achieved in 54 of 80 patients (68%). In 36 of 45 patients (80%) with intact atrioventricular conduction and narrow QRS, an LBB potential (LBB_pot_) was present with an LBB_pot_ to onset of QRS interval of 22 ± 6 ms. QRS duration increased significantly more during RVSP (141 ± 20 ms) than during LBBAP (125 ± 19 ms), compared to 130 ± 30 ms without pacing. An even clearer difference was observed for QRS area, which increased significantly more during RVSP (from 32 ± 16 µVs to 73 ± 20 µVs) than during LBBAP (41 ± 15 µVs). QRS area was significantly smaller in patients with LBB capture compared to patients without LBB capture (43 ± 18 µVs vs 54 ± 21 µVs, respectively). In patients with LBB capture (*n* = 54), the interval from the pacing stimulus to R‑wave peak time in lead V6 was significantly shorter than in patients without LBB capture (75 ± 14 vs 88 ± 9 ms, respectively).

**Conclusion:**

LBBAP is a safe and feasible technique, with a clear learning curve that seems to flatten after 40–60 implantations. LBB capture is achieved in two-thirds of patients. Compared to RVSP, LBBAP largely maintains ventricular electrical synchrony at a level close to intrinsic (narrow QRS) rhythm.

**Supplementary Information:**

The online version of this article (10.1007/s12471-022-01679-7) contains supplementary material, which is available to authorized users.

## What’s new?

- This is the first publication describing detailed left bundle branch area pacing (LBBAP) implantation characteristics of patients receiving their first pacemaker in the Netherlands.
- There is a learning curve for LBBAP implantation, even in a centre with cardiologists experienced with His bundle pacing, which flattens after 40–50 procedures.
- Although QRS duration remains prolonged, LBBAP largely maintains left ventricular synchrony at values close to intrinsic sinus rhythm with normal atrioventricular conduction and narrow QRS.
- In patients with left bundle branch block, LBBAP reduces QRS duration and normalises the QRS vector in the transverse plane.
- QRS area and V6 R‑wave peak time are significantly shorter during LBBP compared to left ventricular septal pacing.

## Introduction

Right ventricular (RV) pacing is a frequently applied therapy in patients without a reversible cause of bradyarrhythmia. RV apex (RVA) pacing produces a non-physiological activation sequence [1], which can lead to adverse remodelling potentially inducing atrial fibrillation, heart failure and cardiovascular death [2, 3].

In the search for a therapy which avoids these detrimental effects of artificial stimulation, there is increasing interest in pacing techniques that directly activate the specialised conduction system. One of these so-called conduction system pacing (CSP) techniques is His bundle pacing (HBP). Since the first application of permanent HBP by Deshmukh and colleagues [4] HBP has proved to be a safe and feasible technique, especially in patients requiring treatment for bradyarrhythmia [5, 6]. HBP involves technical challenges, such as high and unstable pacing thresholds in some patients and relatively low R‑wave amplitude, complicating pacemaker programming [7, 8]. This seems to have limited widespread application in routine clinical practice. As with all CSP strategies, implantation requires more technical skills compared to RV pacing.

An alternative to HBP is left bundle branch area pacing (LBBAP). After it was previously shown that it is possible to reach the left side of the interventricular septum (IVS) [9, 10], it was more recently shown that it is even possible to capture the left bundle branch (LBB) with the same pacing electrode that is currently mostly used for HBP. LBBAP seems to have the advantage of overcoming some of the limitations of HBP while preserving activation of the specialised conduction system.

The aim of our study was (1) to prospectively evaluate the feasibility and learning curve of LBBAP implantation, in a specialised centre with previous experience of ~30 permanent HBP implantations but without previous LBBAP experience; (2) to demonstrate the level of electrical synchrony produced by LBBAP; and (3) to evaluate current LBB capture criteria in patients undergoing pacemaker implantation for either bradycardia treatment or as a bail-out strategy in the case of failed left ventricular (LV) lead implantation.

## Methods

The study was performed at Maastricht University Medical Centre (MUMC+, Maastricht, The Netherlands) in patients undergoing attempted LBBAP for either bradycardia treatment or as a bail-out strategy in the case of failed LV lead implantation. The local ethics committee and Institutional Review Board approved the study protocol (METC 2019–1313) and all patients provided written informed consent. Patients were prospectively enrolled from December 2019 until December 2020.

### Patient selection

All patients undergoing permanent pacemaker implantation who underwent attempted LBBAP were included. Patients underwent LBBAP because of bradycardia (sinus node dysfunction or atrioventricular (AV) block), as part of an ablate and pace strategy for permanent atrial fibrillation, and LBBAP was attempted in some patients with an indication for cardiac resynchronisation therapy (CRT) if previous implantation of the LV lead or His bundle lead had failed.

### Implantation procedure

The LBBAP implantation procedure was performed as described previously [11]. In brief, the right atrial (RA) lead (if implanted) was implanted according to routine clinical practice. In the case of underlying left bundle branch block (LBBB), the RA lead was temporarily placed in the right ventricle, ensuring the possibility of back-up pacing in case of manipulation-induced total AV block.

The ventricular pacing lead (Medtronic 3830 lead) that was used for LBBAP was positioned using the C315His sheath (Medtronic, Minneapolis, MN, USA) in all patients. An intracardiac electrogram was recorded from the lead tip in a unipolar fashion using an electrophysiological recording system (Labsystem Pro, Boston Scientific, Marlborough, MA, USA). First, the His bundle electrogram was identified in the right anterior oblique 20–25° position and a fluoroscopic image was recorded as a reference (Electronic Supplementary Material, Fig. S1, upper left). Subsequently, the sheath and lead were advanced 1–2 cm toward the RVA (Electronic Supplementary Material, Fig. S1, upper right). In this region, unipolar pacing was performed aiming for a paced QRS morphology with a notch in the nadir of lead V1, resembling a ‘W’ (Electronic Supplementary Material, Fig. S2a). Alongside this notched QRS complex in lead V1, a positive QRS complex in lead II and negative complex in lead III are good indicators of an appropriate position. At this site, the lead was fixed in the RV septum with 1–2 rotations and then advanced to the left side of the IVS in a left anterior oblique view (Electronic Supplementary Material, Fig. S2b–e). In the process of advancing the pacing lead, fluoroscopy, pacing threshold and lead impedance, the paced QRS morphology, and the presence and morphology of fixation beats were monitored to estimate the depth of the lead, avoiding perforation of the IVS [12].

The number of attempts to implant the lead in the IVS as well as the final position were left to the discretion of the implanting cardiologist. Capture of the LBB (trunk or proximal fascicles) was attempted in all patients.

### Pacing and capture definitions

LBB capture can be demonstrated through several mechanisms, such as the presence of a transition from myocardial capture to conduction system capture (or vice versa) during threshold testing, by measuring the interval between LBB potential and R‑wave peak time in lead V6 (V6RWPT) [13] or through programmed stimulation [14] or making use of the difference in effective refractory period between myocardium and the specialised conductive tissue.

Other common characteristics of LBB pacing are: (1) paced (pseudo) right bundle branch block (RBBB) QRS morphology with a terminal R/R′ wave in lead V1; (2) recording of an LBB potential during intrinsic rhythm (only in patients with intact AV conduction); (3) constant stimulus to V6RWPT during high (8 V) and low (2 V) pacing output.

In our study, LBB capture was diagnosed if one (or both) of the following criteria were met: (1) the presence of a transition from non-selective LBBP (nsLBBP) to selective LBBP (sLBBP) or from nsLBBP to LV myocardial only capture (=LVSP, LV septal pacing) during decreasing pacing output; (2) LBB potential to V6RWPT interval equal to the pacing stimulus to V6RWPT interval [13].

sLBBP was defined as a change in QRS morphology without a change in V6RWPT when decreasing the pacing output from nsLBBP, combined with a pacing artefact distinct from the ventricular electrogram. nsLBBP was defined as a change in QRS morphology which occurred after increasing the pacing output from sLBBP or LVSP. LVSP was defined as paced QRS morphology with an R′ wave present in lead V1 but without evidence of LBB capture.

### Electrical measurements

Twelve-lead electrocardiograms (ECGs) were recorded during intrinsic rhythm, right ventricular septal pacing (RVSP) and LBBAP. QRS duration was measured from onset of first deflection, thus excluding the pace spike. In the presence of an LBB potential, the interval between the LBB potential and QRS onset as well as the interval between the LBB potential and V6RWPT were measured.

Fixation beats are defined as ectopic ventricular beats resulting from irritation of tissue as the lead crosses the septum. Ectopic beats with QRS complexes < 120 ms with qR/rsR’ morphology in lead V1 are considered to be beats from the LBB area.

QRS area, a measure of ventricular electrical dyssynchrony [15], was determined by converting the two-dimensional ECG into a three-dimensional vectorcardiogram (VCG). The VCG was synthesised as described previously [15, 16]. In brief, the original digital signals were extracted from the ECG files stored in the BARD system. Subsequently, custom Matlab software (MathWorks Inc, Natick, MA, USA) was used to convert the 12-lead ECG into the three orthogonal vectorcardiographic leads (X, Y, and Z) using the Kors conversion matrix [17]. QRS area was calculated as the sum of the area under the QRS complex in the calculated vectorcardiographic X, Y, and Z leads:

$$[\text{QRS area}=\left({\mathrm{QRS}_{\text{area},\mathrm{x}}}^{2}+{\mathrm{QRS}_{\text{area},\mathrm{y}}}^{2}+{\mathrm{QRS}_{\text{area},\mathrm{z}}}^{2}\right)^{1/2}].$$

### Data collection

The demographic data and medical history of all patients were collected at enrolment. Procedure-related characteristics, including ECG characteristics and intracardiac electrogram patterns, fluoroscopy exposure time and doses, were recorded during implantation. Pacing parameters (pacing threshold, pacing impedance, and R‑wave amplitude) were measured immediately post-implantation and up to 1‑year follow-up.

### Safety endpoints

Data on major acute procedure-related adverse events such as bleeding, pneumo- and haemothorax, and cardiac tamponade were collected. Also, device and lead-related problems such as infection, perforation, dislodgement or dysfunction at any time during follow-up were recorded. Adverse event treatment was classified as re-intervention, prolonged hospitalisation or death.

### Statistical analysis

The number and percentage were used as descriptive statistics for categorical variables. Continuous variables are expressed as mean ± standard deviation. Differences between two groups were compared using the Student *t*-test for continuous variables. The paired *t*-test was used to compare the differences between two means within the same group. Comparisons among ≥ 3 pacing conditions within individuals were made using repeated measures analysis of variance with the Bonferroni multiple comparisons procedure applied to pairwise comparisons. A two-sided *p*-value of < 0.05 was considered statistically significant. All statistical analyses were performed using SPSS Statistics version 25.0 (IBM Corp., Chicago, IL, USA).

## Results

### Baseline characteristics

Eighty patients underwent permanent pacemaker implantation with attempted LBBAP. Patient characteristics are summarised in Tab. 1. Mean age was 74 ± 10 years and 59% of patients were men. A history of hypertension was recorded in 58% and coronary artery disease was present in 38% of patients. LV ejection fraction (LVEF) at baseline was 53 ± 10% with an LVEF < 50% in 18 of 80 patients (23%). The indication for pacemaker implantation was sinus node dysfunction in 27 patients (35%), AV block in 32 patients (41%), AV-node ablation in 8 patients (10%), and CRT in 10 patients (13%).

**Table 1:** Baseline characteristics of the study population

Characteristics (*n* = 80)	Mean ± SD or *n* (%)
Male sex	47 (59%)
Age (years)	74 ± 10
*Medical history*	
– Hypertension	46 (58%)
– Diabetes mellitus	16 (20%)
– Atrial fibrillation	33 (41%)
– Coronary artery disease	30 (38%)
– LVEF < 50%	18 (23%)
*Echocardiographic parameters*	
– LVEF (%)	53 ± 10
– LV end-diastolic diameter (mm)	51 ± 7
– LV end-systolic diameter (mm)	37 ± 7
– IVS thickness (mm)	10 ± 1
*Electrocardiographic parameter*s	
– Heart rate (bpm)	67 ± 20
– QRS duration (ms)	
a. all patients	116 ± 31
b. intrinsic ventricular conduction	95 ± 13 (*n* = 45)
c. Other (escape, LBBB/RBBB, paced)	143 ± 26 (*n* = 35)
*Pacemaker indication*	
– Sinus node dysfunction	27 (35%)
– Atrioventricular block	32 (41%)
– Atrial tachyarrhythmia requiring ablation	8 (10%)
– Heart failure and prolonged QRS duration	10 (13%)

*LV* left ventricular, *LVEF* LV ejection fraction, *IVS* interventricular septum, *LBBB* left bundle branch block, *RBBB* right bundle branch block

### Procedural characteristics

All implantation procedures were performed using the C315His delivery catheter (Medtronic) and the SelectSecure 3830 lead (Medtronic). In 75 of 80 patients (94%) a de novo pacemaker implantation was performed. Permanent LBBAP lead implantation was successful in 77 of 80 patients (96%). In two patients with concentric LV hypertrophy, RV dilatation and known coronary artery disease, the ventricular lead could, not be advanced deep enough into the septum, despite multiple attempts, resulting in a broad paced QRS duration without evidence of at least deep (LV) septal pacing. In these patients, the lead was then positioned in the apico-septal region of the right ventricle. In one patient with dilated right atrium, right and left ventricle, no stable LBBAP position could be achieved, and this patient was converted to HBP, whereby selective His capture was achieved.

**Fig. 1:**
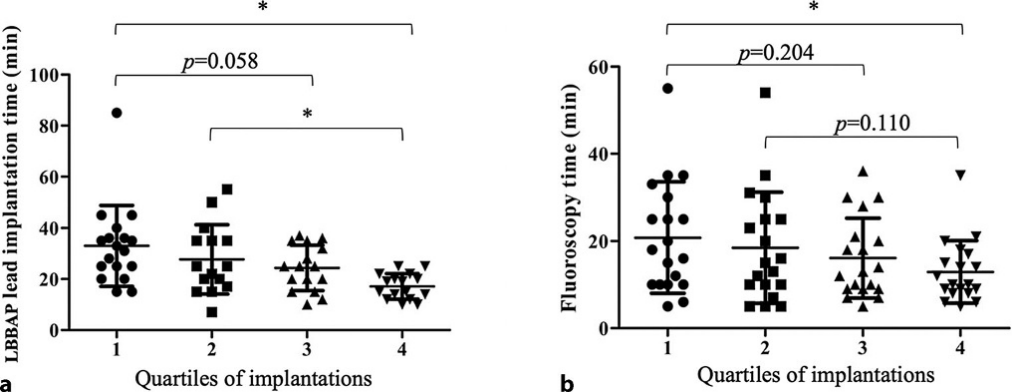
Comparison of the duration of left bundle branch area pacing (*LBBAP*) lead implantation (**a**) and fluoroscopy time (**b**) in four quartiles of implantations

Total procedure time, defined as time from first incision to last suture, was 86 ± 31 min and LBBAP lead implantation time was 25 ± 13 min. The mean radiation time and dosage across all procedures was 17 ± 11 min and 97 ± 65 mGy, respectively. Implantation procedure times reduced with increasing LBBAP experience, as shown in Fig. 1. The implantation time of the LBBAP lead decreased considerably from 33 ± 15 min during the initial implantations to 17 ± 5 min during the more recent implantations. Also, the associated fluoroscopy time was reduced significantly from 21 ± 12 min to 13 ± 7 min (Fig. 1b), illustrating the learning curve of LBBAP implantation.

### Electrocardiographic characteristics

LBB capture, according to previously described criteria, was confirmed in 54 of 80 patients (68%). In patients with LBB capture, a transition from nsLBBP to sLBBP or vice versa (Electronic Supplement Material, Fig. S3a) could be demonstrated in 37 of 54 patients (69%) during threshold testing. In 17 of 54 patients (31%), a transition from nsLBBP to LVSP was observed (Electronic Supplement Material, Fig. S3b). Out of 80 patients, 45 had intact AV conduction with a narrow intrinsic QRS complex. In 36 of 45 patients (80%) a clear LBB potential was present. The interval between the LBB potential and the onset of QRS was 22 ± 6 ms.

For all patients, QRS duration increased from 130 ± 30 ms during intrinsic rhythm to 141 ± 20 ms during RVSP and decreased to 125 ± 19 ms during LBBAP. Final QRS duration was 124 ± 20 ms in patients where LBB capture was achieved, and 130 ± 24 ms in patients with LVSP (without LBB capture; *p* = 0.397). QRS area significantly increased from 49 ± 35 µVs during intrinsic rhythm to 80 ± 22 µVs during RVSP. Compared to RVSP, QRS area decreased to 48 ± 19 µVs during LBBAP.

**Fig. 2:**
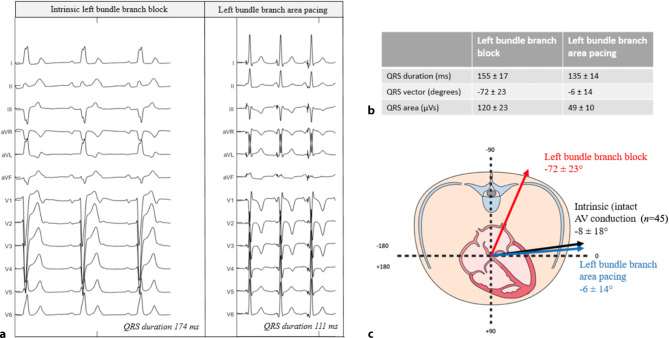
**a–c** Electrical characteristics of left bundle branch block (LBBB) patients. **a** Example of a 12-lead electrocardiogram during intrinsic LBBB (*left*) and left bundle branch area pacing (*right*) in the same patient. **b** Vectorcardiographic results of all LBBB patients (*n* = 7). **c** Schematic overview of the heart in the transverse plane with QRS vector of patients during LBBB and left bundle branch area pacing (*n* = 7), and QRS vector during intrinsic sinus rhythm in patients with normal conduction (*n* = 45)

Out of 80 patients, 35 had a broad intrinsic QRS complex (18 escape rhythm, 7 complete LBBB, 3 left anterior fascicular block, 1 left posterior fascicular block, 4 RBBB, 2 RV paced). In patients with complete LBBB, QRS duration was significantly reduced from 155 ± 17 ms during intrinsic rhythm to 135 ± 14 ms during LBBAP, while QRS area was significantly reduced from 120 ± 23 µVs during intrinsic LBBB to 49 ± 10 µVs during LBBAP (Fig. 2).

**Fig. 3:**
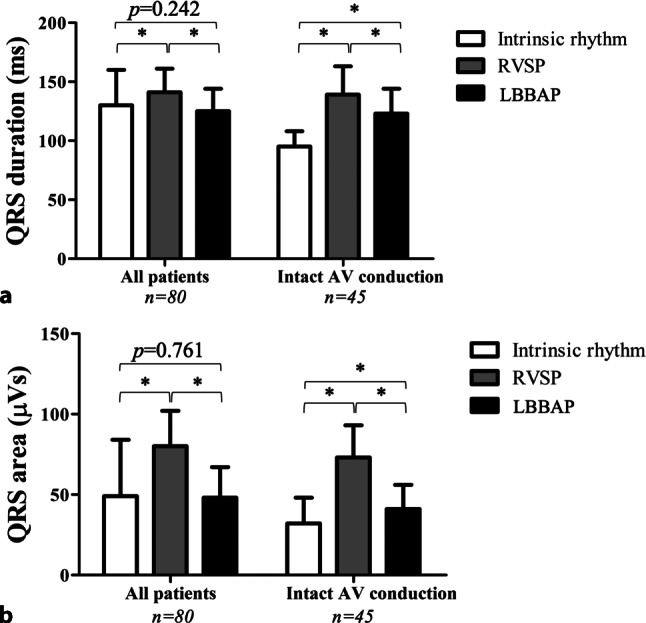
**a**, **b** QRS duration and QRS area. QRS duration (**a**) and QRS area (**b**) during intrinsic rhythm, right ventricular septum pacing (*RVSP*) and left bundle branch area pacing (*LBBAP*) in all patients and in the subpopulation with intact atrioventricular activation and a narrow QRS complex. *AV* atrioventricular. *p < 0.05 between pacing modes

In patients with intact AV conduction and a narrow intrinsic QRS complex (*n* = 45), QRS duration significantly increased during both RVSP and LBBAP (Fig. 3a). QRS area significantly increased during both RVSP and LBBAP, although QRS area during LBBAP approached QRS area values during intrinsic rhythm (Fig. 3b). QRS area was significantly smaller in patients with LBB capture compared to those without capture (43 ± 18 vs 54 ± 21 µVs, respectively).

During LBBAP, V6RWPT was 82 ± 13 ms. In patients with LBB capture (*n* = 54), V6RWPT was significantly shorter compared to those without LBB capture (75 ± 14 ms vs 88 ± 9 ms, respectively). Four patients with LBB capture had a long V6RWPT with an interval > 100 ms. These patients had a long iso-electric segment (> 30 ms) with left axis deviation, suggesting a proximal conduction delay.

### Fixation beats

Fixation beats have been suggested to be of help in determining lead depth when the lead is advanced into the septum [12]. In 54 of the 80 patients in our study (67%) ventricular ectopic beats (deep septal fixation beats) were observed. Figure S4 in the Electronic Supplement Material shows that these fixation beats closely resembled the paced morphology obtained at that particular intermediate lead depth in the IVS.

### Pacing characteristics

The unipolar LBBP lead threshold was 0.6 ± 0.3 V at 0.5 ms pulse width at implantation, as measured by the programmer. The sensed R‑wave amplitude and pacing impedance at implantation were 14 ± 8 mV and 605 ± 212 Ω, respectively.

The unipolar pacing threshold did not significantly change from time of implantation (*n* = 80; 0.6 ± 0.3 V) to 3‑month (*n* = 70; 0.6 ± 0.2 V), 6‑month (*n* = 55; 0.7 ± 0.3 V) or 12-month follow-up (*n* = 40; 0.8 ± 0.5 V). The sensed R‑wave amplitude remained stable at 12-month follow-up compared to time of implantation (18 ± 9 mV vs 14 ± 8 mV, not significant). Pacing impedance significantly changed from 605 ± 212 Ω to 365 ± 42 Ω at 12-month follow-up (*p* < 0.05).

### Safety endpoints

Peri-procedurally, no major acute procedure-related adverse events such as bleeding, pneumothorax or haemothorax or cardiac tamponade occurred. Acute perforation of the LV septum was noted in one patient during implantation. Retrospective analysis of the procedure revealed (missed) rapidly appearing fixation beats occurring at the very end of septal penetration, which indicated that the LBB area was reached. In this case, the lead was withdrawn and repositioned successfully. Post-procedural echocardiography with colour Doppler revealed no complications from this temporary septal perforation. One patient with pre-existing serious coronary artery disease (three-vessel disease) experienced an acute coronary syndrome and in-hospital cardiac arrest (occluded D1 branch of left anterior descending artery) during implantation, for which an urgent percutaneous coronary intervention (PCI) was performed. Chest complaints accompanied by minor ECG changes started prior to septal penetration and no contrast agent was used. After consultation with the intervention specialist, no acute coronary angiography was performed since the patient suffered from extensive three-vessel disease (and conservative treatment was previously decided upon). Sublingual administration of nitroglycerin initially relieved complaints, but during lead placement the patient went into cardiogenic shock. Urgent PCI was successful and the patient is now participating in the cardiac rehabilitation programme.

During follow-up, lead dislodgement during follow-up was observed in one patient, for which lead repositioning was performed. No device or lead infections were observed.

## Discussion

The main findings of our study are as follows:

1. Permanent LBBAP as a new physiological pacing technique is feasible (96% success rate) and safe, as it is not associated with significant adverse effects.
2. There is a learning curve for LBBAP implantation, even in a centre with cardiologists experienced in HBP lead implantation, which flattens after 40–50 procedures.
3. The electrical characteristics of the LBBAP lead are satisfying and remain stable over time.
4. LBBAP results in LV synchrony, measured by QRS area, which is significantly better than RV pacing and approximates that of intrinsic rhythm in patients with intact AV conduction and a narrow QRS.

### Safety and feasibility

The possibility of penetrating the IVS to obtain more synchronous pacing was previously shown by our group and referred to as LVSP. The feasibility of permanent LVSP was first shown in a canine model [9] and later also in patients requiring pacemaker implantation because of symptomatic bradycardia [10]. In both animal experiments and in patients it was shown that LVSP resulted in improved cardiac function when compared to RV pacing. In these studies, the lead was placed at the mid-level of the IVS and direct capture of the conduction system was not studied or pursued. After these initial studies, it was demonstrated only very recently that the LBB can be stimulated when the lead is advanced through the IVS at a basal level [18]. Since then, more studies including bradycardia patients as well as CRT patients have demonstrated the feasibility and safety of LBBAP [19–21]. Our results, representing experience at a single centre where this new pacing technique was initiated, are in line with these studies. In the present study, LBBAP was attempted in 80 patients and it was possible to achieve LBBAP in 96% of the patients without any major procedure-related adverse events. We did observe one septal perforation, which occurred without any clinical consequences. The results of the study are in line with those of recent large-scale prospective registries [22, 23]. Compared to the results from the USA and China, success rate (MUMC +96%, China 98%, USA 89%), procedure time (MUMC +86 ± 31 min, China N/A, USA 75 ± 34 min) and fluoroscopy time (MUMC +10 ± 8 min, China N/A, USA 10 ± 8 min) were comparable. Also, the presence of an LBB potential (MUMC +45%, China 77%, USA 41%) and the LBB capture rate (MUMC +68%, China 70%, USA 55%) are comparable.

### Learning curve

The present study demonstrated that LBBAP implantation is subject to a clear learning curve effect, even for cardiologists experienced in HBP implantation. Although the implantations are performed by more than one cardiologist, we chose to demonstrate one general learning curve as implantations are performed by two cardiologists who performed implantations alternately.

With increasing experience, procedure and lead implantation time shortened, which has also been demonstrated for HBP [24]. In our centre, all implantations were performed using the C315His delivery sheath and the SelectSecure 3830 pacing lead (both Medtronic). Although the 3830 lead is not intended exclusively for LBBAP, it is the most frequently used lead in LBBAP, since the screw tip is the active electrode so that pacing can be delivered precisely and the lead depth in the IVS can be determined with relative precision. Procedure success, specifically LBB capture rate, could potentially be improved with newer, dedicated materials from different vendors. Especially in patients with dilated ventricles, the currently available delivery catheters do not usually suffice.

The LBB capture rate can also be related to the learning curve and implantation experience. An experienced implanting cardiologist is potentially less afraid of penetrating the septum and therefore more confident while advancing the lead towards the left side of the conduction system, which is situated at the LV subendocardium [25]. Also, rotating the lead without pushing firmly enough can result in a so-called drill effect, causing the lead not to advance deep enough and to lack stability.

### Assessing septal lead depth

Determining the exact depth of the pacing lead within the septum often remains difficult. Several manoeuvres to monitor lead depth have been proposed, such as fluoroscopy imaging (e.g. fulcrum sign), with or without septal contrast, impedance monitoring, or monitoring of the endocardial signal. These manoeuvres are useful but usually do not suffice. The paced QRS morphology can be used, since an RBBB-like QRS morphology indicates left-sided IVS pacing [26]. It was recently showed that the appearance of an R′ wave in lead V1 during LBBAP corresponds to a small QRS area, indicating a low level of LV intraventricular dyssynchrony [27]. While with conventional connector cables it is usually not possible to perform ventricular pacing during screwing, recently investigated so-called fixation beats are particularly useful. These ventricular ectopic beats become apparent as a result of screwing in the lead and are identical to the paced QRS morphology [12]. While in the original publication these fixation beats were present in 96% of the cases, we found these beats in only 67% of implantations. This difference may be primarily due to the difference in definition: we considered only ectopic beats with qR/rsR’ morphology in lead V1 to be beats from the LBB area, while in the original publication ectopic beats without an R wave in V1 were also considered to originate from the LBB area when the QRS duration was < 130 ms. As new implantation tools become available with stylet-driven leads, continuous pacing is an option as an alternative to fixation beats. Lastly, V6RWPT can be used, since V6RWPT (~ LV activation time) shortens during left-sided IVS pacing compared to right-sided pacing [27]. Instead of waiting for mechanically induced ectopic beats, local depolarisations can also be forced by continuous pacing during the whole process of lead rotation/implantation. During lead progression from the right to the left side of the septum the paced QRS changes: the QRS gradually narrows, the R wave appears in V1 and V6RWPT shortens [28].

### Capture criteria

Confirmation of LBB capture is challenging. A recently proposed indirect measurement that could help to identify LBB capture is the interval between the LBB potential and V6RWPT during native conduction as compared to the V6RWPT during pacing [29]. The difference (∆) in V6RWPT during HBP and nsLBBP/LVSP can be used to assess LBB capture in CRT patients [30]. Furthermore, the V6-V1 interpeak interval can differentiate the three types of LBB area capture: non-selective LBB, selective LBB, and LV septal capture [31].

In our study, we used the criteria proposed by Huang et al. [11] with the addition that a transition of nsLBBP to sLBBP or nsLBBP to LVSP is required. This in order to be able to differentiate LBB capture from LV septum only pacing.

A different method of establishing whether the LBB has been captured might be by measuring His-Purkinje potentials, especially in patients without pre-existing bundle branch block. Using electrophysiology catheters along the left side of the IVS and along the bundle of His would allow recording of retrograde His potentials (with a short stimulus-His interval) or anterograde LBB potentials to differentiate between deep LV septal pacing and LBBP. However, performing these kinds of measurements would increase the invasiveness of the procedure, as well as costs, and is therefore not appropriate for routine use in daily clinical practice. However, future studies using these invasive electrophysiological measurements would be of interest, as they would increase our mechanistic insight into LBBAP. Establishing LBB capture at this stage of LBBAP is considered important, since we are as yet unaware of certain patient (sub)populations that benefit from either LVSP or LBBP. If long-term outcome does not differ between the two, the advanced measurements and implantation associated with LBBP might even be redundant.

### Electrophysiological effects

QRS duration significantly increased during RVSP in our population and remained prolonged during LBBAP when compared to intrinsic normal ventricular activation in patients with intact AV conduction. This finding is in agreement with previous studies investigating LBBAP [32, 33]. A prolonged QRS duration is to be expected in RVSP, since the His-Purkinje system is not recruited. In LBBAP, the prolonged QRS duration is mainly due to a delayed RV activation, while LV activation is restored. This delayed RV activation becomes evident on the ECG as an R′ wave in V1. The haemodynamic and long-term effect of this delayed RV activation caused by LBBAP needs to be carefully evaluated in future studies.

In contrast to QRS duration, the QRS area normalised to a large extent during LBBAP in the present study. The QRS area, which is calculated after converting the standard 12-lead ECG, serves as a measure of ventricular electrical dyssynchrony [15]. In previous studies investigating CRT, it was shown that the QRS area has a strong association with clinical and echocardiographic response [34]. In a more recent study by our group, it was even shown that the decrease in QRS area after CRT was a strong independent predictor of echocardiographic and clinical CRT response [35]. In the present study, both LBBP and LVSP resulted in a small QRS area, approximating the intrinsic QRS area in patients with intact AV conduction and a narrow QRS. LBBP resulted in a significantly smaller QRS area as compared to LVSP, although the difference in the absolute value is small. Whether this small difference between LBBP and LVSP results in different clinical outcomes needs to be determined.

### Limitations

Our study shows the results of a prospective registry evaluating the feasibility and electrical characteristics of LBBAP performed by operators (JL, BW, KV) with experience of ~30 permanent HBP implantations before starting with LBBAP. The majority of implantations were performed alternately by two operators. Our study demonstrates a learning curve in a limited number of patients and analysis of larger numbers is required to validate our findings. LVSP was accepted somewhat faster in the initial cases, which could have influenced the LBB capture rate. With growing experience in LBBAP implantations and increasing knowledge of LBBAP, especially as regards lead depth in the interventricular septum, implantation skills have probably improved in our centre, which has probably influenced the results. Nevertheless, we are convinced that the data provided in this study represent real-world data of the initial experience in a centre starting with LBBAP.

## Conclusions

LBBAP is a new implantation technique that is feasible and safe in a high percentage of patients. Pacing characteristics are very satisfactory and remain stable during 1‑year follow-up. Capture of the left bundle, defined by strict criteria, could be achieved in up to two-thirds of patients. Although QRS duration remains prolonged, LBBAP largely maintains LV synchrony at a level close to intrinsic sinus rhythm with normal AV conduction and a narrow QRS. New measures to determine LBB capture, such as V6RWPT equals LBB potential to V6RWPT interval, seem promising. Moreover, the QRS morphology of the fixation beats is helpful in determining lead depth while screwing the lead into the interventricular septum.

## Supplementary Information

Fig. 1. Fluoroscopic localization for LBBAP lead implantation. Location of the His bundle potential (*upper left*) and LBBAP lead fixation (*upper right*) in right anterior oblique (RAO) fluoroscopic views are shown. A thin layer of contrast is seen against the right ventricular septal wall (*lower left*) in left anterior oblique (LAO) fluoroscopic view, demonstrating the lead depth in the septum. Final lead position is shown in RAO view (*lower right*).

Fig. 2. Twelve lead electrocardiographic recordings during LBBAP lead implantation at sweep speed of 25 mm/s. Electrocardiograms are shown during various depths of lead fixation. **A**: unipolar pacing produces paced QRS morphology with a notch in the nadir in lead V1 (*circle*) with a positive QRS complex in lead II and negative complex in lead III. **B** and **C**: the lead is driven towards left side of interventricular septum and the notch in lead V1 shifts to the right. **D**: the QS morphology changes to Qr pattern (*circle*), indicating the left side of the septum is reached. **E**: Final fixation. **F**: presence of left bundle potential (*circle*) during intrinsic rhythm (sweep speed 200 mm/s).

Fig. 3A. Transition selective left bundle branch pacing (s-LBBP; first two beats) to non-selective (ns-LBBP; last two beats). Twelve-lead electrocardiograms and intracardiac electrograms leads are shown at sweep speed of 100 mm/s. During unipolar pacing with increasing pacing output, transition of s‑LBBP to ns-LBBP is shown. Note the discrimination of the pace spike from the ventricular EGM in the first two beats (ellipse), which disappears with increased output while RWPT V6 is unchanged. During intrinsic rhythm, a LBB potential is recorded. Fig. 3B. Transition non-selective left bundle branch pacing (LBBP; first two beats) to left ventricular septal capture (LVSP; last two beats). Twelve-lead electrocardiograms and intracardiac electrogram from LBBP lead are shown. Left panel electrocardiograms are shown at sweep speed 100 mm/s, right panel electrocardiograms at sweep speed of 50 mm/s. During unipolar pacing with decreasing pacing output, transition of ns-LBBP to LV septal capture is shown. Note the increase in RWPT V6 of 10 ms and change in QRS morphology (decrease r’ amplitude; circle).

Fig. 4. Electrocardiograms. **A**. 12-lead electrocardiogram during transseptal advancement of the LBBAP lead. Shown are ectopic beats of deep septal origin (“fixation beats”, *circle*), which closely resemble paced morphology obtained at final lead position. Note that the amplitude of the r‑wave of these fixation beats increases during advancement to the subendocardium of the LV. **B**: 12-lead electrocardiogram during LBBAP.
