# Supplementary figures and images for: A single-centre prospective evaluation of left bundle branch area pacemaker implantation characteristics
Source: Neth Heart J. 2022 Apr 5;30(5):249–57. doi: 10.1007/s12471-022-01679-7 (PMC9043076; doi:10.1007/s12471-022-01679-7)

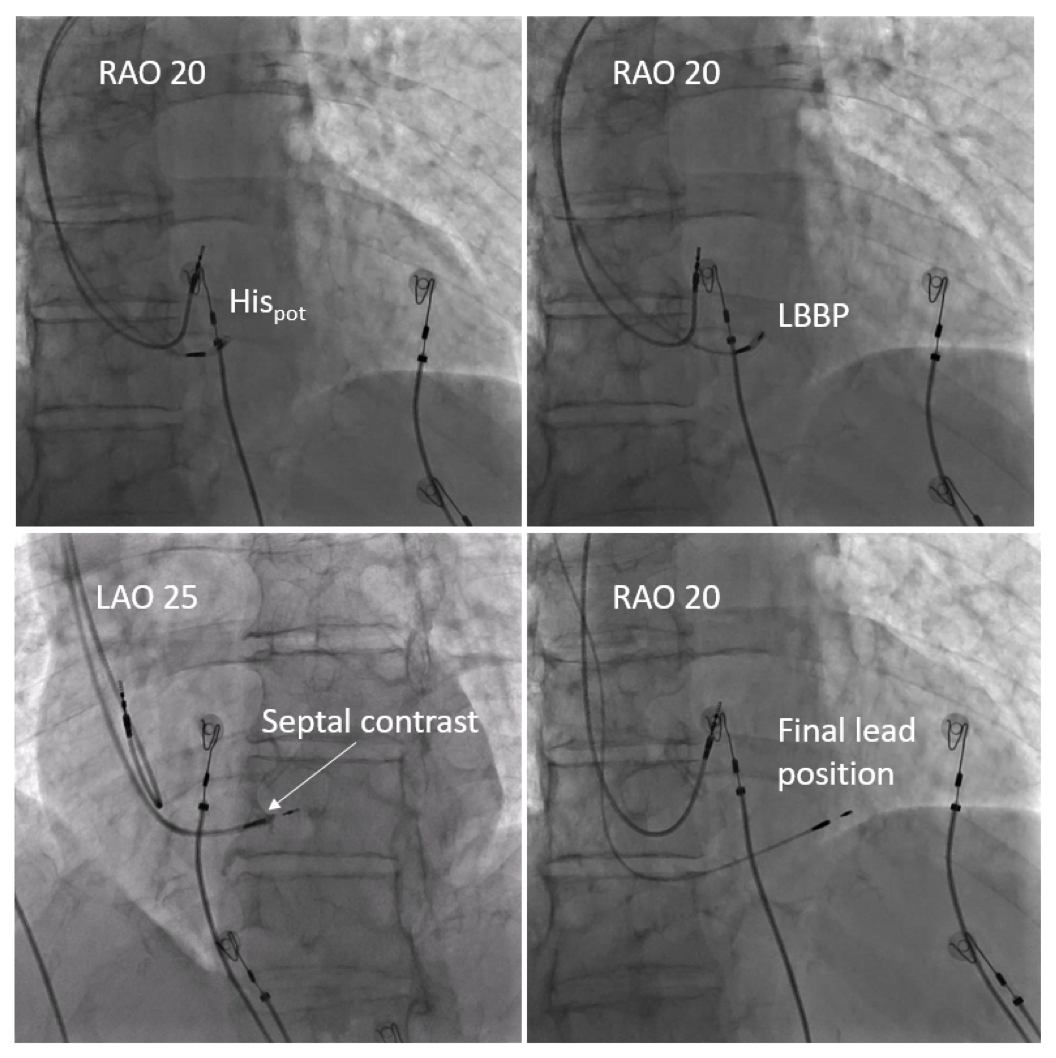

Supplement: Supplementary file 1 — Fig. 1. Fluoroscopic localization for LBBAP lead implantation. Location of the His bundle potential (upper left) and LBBAP lead fixation (upper right) in right anterior oblique (RAO) fluoroscopic views are shown. A thin layer of contrast is seen against the right ventricular septal wall (lower left) in left anterior oblique (LAO) fluoroscopic view, demonstrating the lead depth in the septum. Final lead position is shown in RAO view (lower right). [file 12471_2022_1679_MOESM1_ESM.tiff]

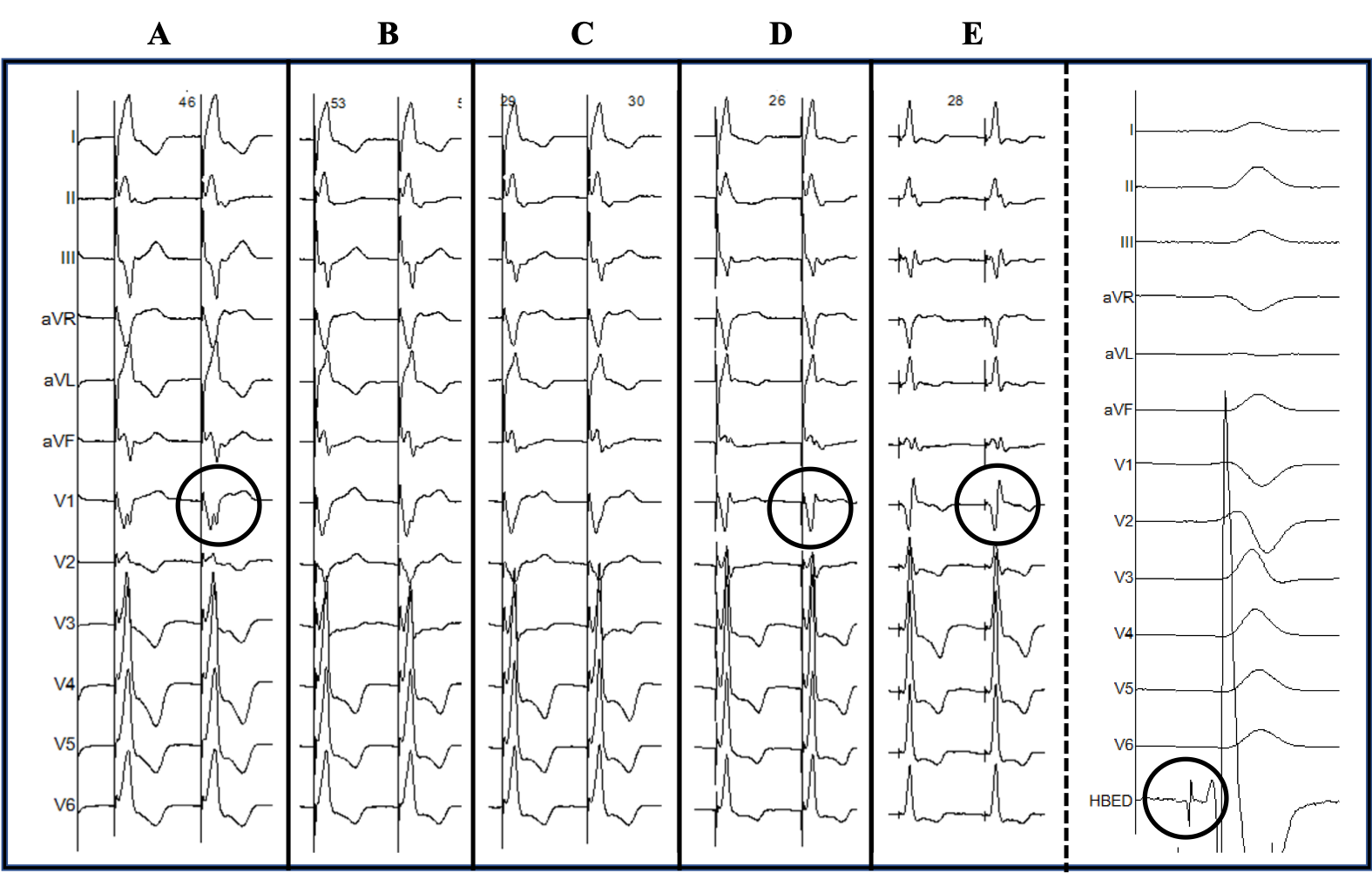

Supplement: Supplementary file 2 — Fig. 2. Twelve lead electrocardiographic recordings during LBBAP lead implantation at sweep speed of 25 mm/s. Electrocardiograms are shown during various depths of lead fixation. A: unipolar pacing produces paced QRS morphology with a notch in the nadir in lead V1 (circle) with a positive QRS complex in lead II and negative complex in lead III. B and C: the lead is driven towards left side of interventricular septum and the notch in lead V1 shifts to the right. D: the QS morphology changes to Qr pattern (circle), indicating the left side of the septum is reached. E: Final fixation. F: presence of left bundle potential (circle) during intrinsic rhythm (sweep speed 200 mm/s). [file 12471_2022_1679_MOESM2_ESM.tiff]

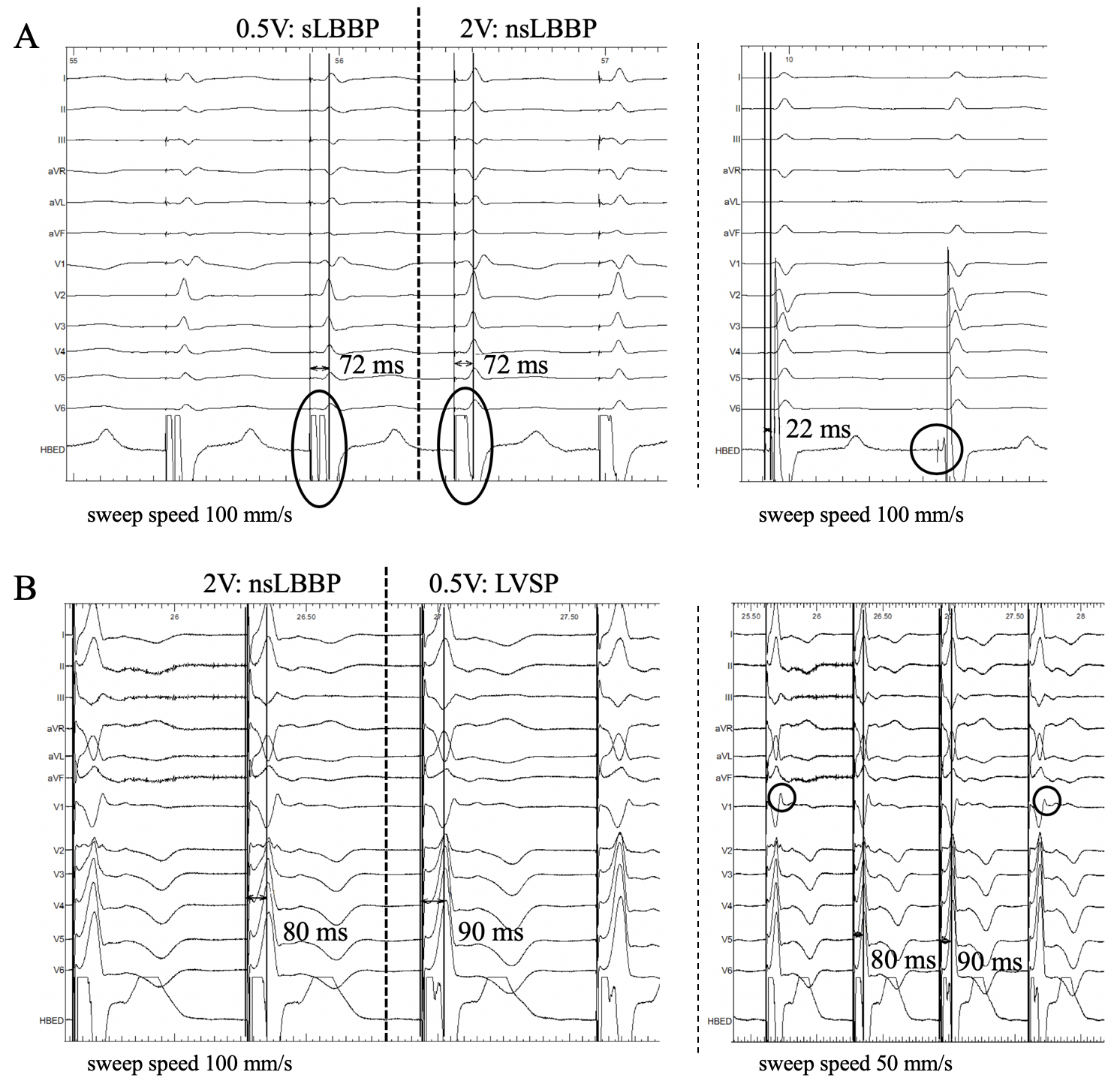

Supplement: Supplementary file 3 — Fig. 3A. Transition selective left bundle branch pacing (s-LBBP; first two beats) to non-selective (ns-LBBP; last two beats). Twelve-lead electrocardiograms and intracardiac electrograms leads are shown at sweep speed of 100 mm/s. During unipolar pacing with increasing pacing output, transition of s‑LBBP to ns-LBBP is shown. Note the discrimination of the pace spike from the ventricular EGM in the first two beats (ellipse), which disappears with increased output while RWPT V6 is unchanged. During intrinsic rhythm, a LBB potential is recorded. Fig. 3B. Transition non-selective left bundle branch pacing (LBBP; first two beats) to left ventricular septal capture (LVSP; last two beats). Twelve-lead electrocardiograms and intracardiac electrogram from LBBP lead are shown. Left panel electrocardiograms are shown at sweep speed 100 mm/s, right panel electrocardiograms at sweep speed of 50 mm/s. During unipolar pacing with decreasing pacing output, transition of ns-LBBP to LV septal capture is shown. Note the increase in RWPT V6 of 10 ms and change in QRS morphology (decrease r’ amplitude; circle). [file 12471_2022_1679_MOESM3_ESM.tiff]

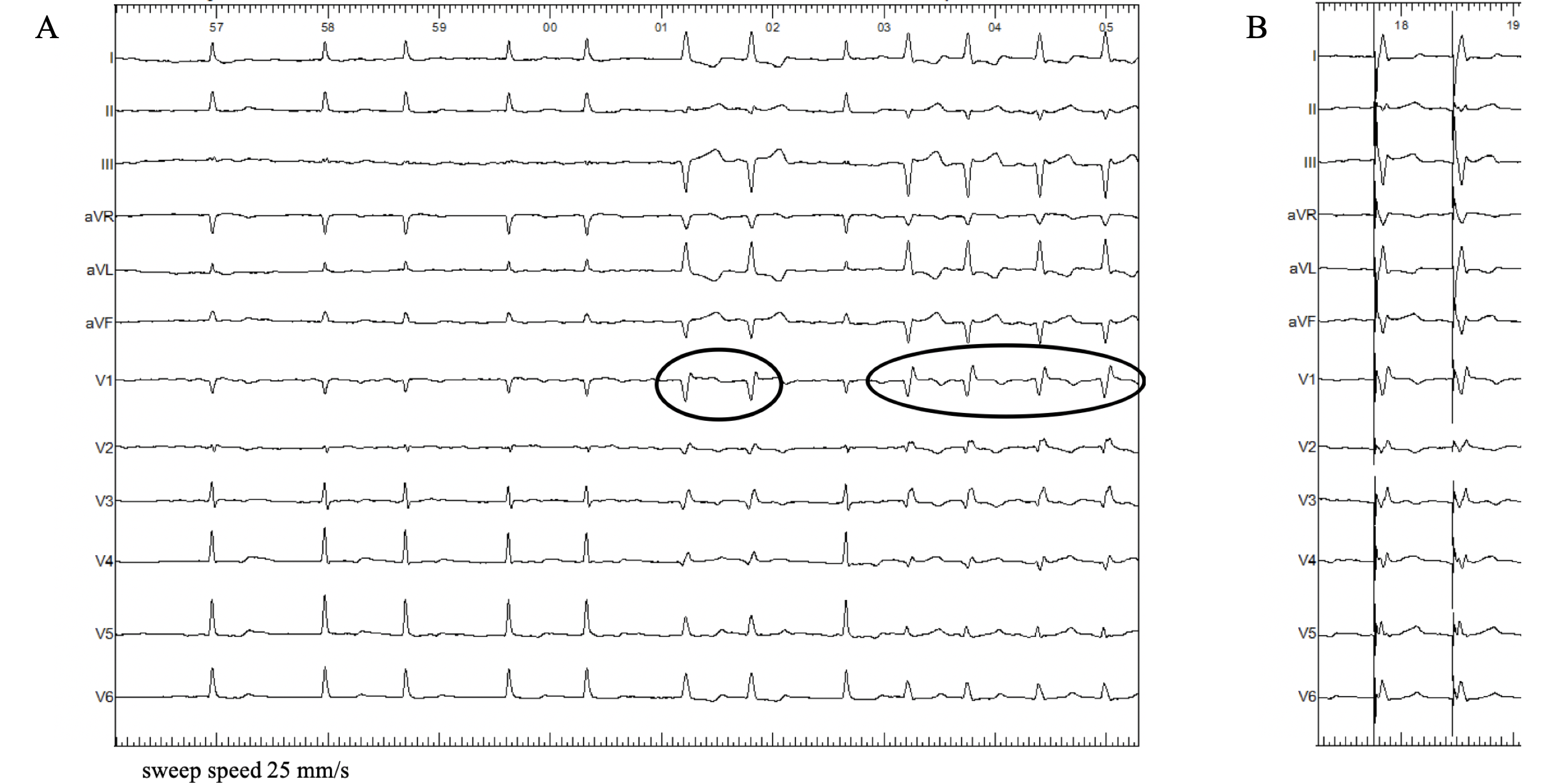

Supplement: Supplementary file 4 — Fig. 4. Electrocardiograms. A. 12-lead electrocardiogram during transseptal advancement of the LBBAP lead. Shown are ectopic beats of deep septal origin (“fixation beats”, circle), which closely resemble paced morphology obtained at final lead position. Note that the amplitude of the r‑wave of these fixation beats increases during advancement to the subendocardium of the LV. B: 12-lead electrocardiogram during LBBAP. [file 12471_2022_1679_MOESM4_ESM.tiff]
